# Social learning within and across predator species reduces attacks on novel aposematic prey

**DOI:** 10.1111/1365-2656.13180

**Published:** 2020-02-19

**Authors:** Liisa Hämäläinen, Johanna Mappes, Hannah M. Rowland, Marianne Teichmann, Rose Thorogood

**Affiliations:** ^1^ Department of Zoology University of Cambridge Cambridge UK; ^2^ Department of Biological and Environmental Sciences University of Jyväskylä Jyväskylä Finland; ^3^ Max Planck Institute for Chemical Ecology Jena Germany; ^4^ Institute of Zoology Zoological Society of London London UK; ^5^ HiLIFE Helsinki Institute of Life Science University of Helsinki Helsinki Finland; ^6^ Research Programme in Organismal & Evolutionary Biology Faculty of Biological and Environmental Sciences University of Helsinki Helsinki Finland; ^7^ Chair of Nature Conservation & Landscape Ecology University of Freiburg Freiburg Germany

**Keywords:** aposematism, avoidance learning, conspecific information, heterospecific information, predator–prey interactions, social learning

## Abstract

1. To make adaptive foraging decisions, predators need to gather information about the profitability of prey. As well as learning from prey encounters, recent studies show that predators can learn about prey defences by observing the negative foraging experiences of conspecifics. However, predator communities are complex. While observing heterospecifics may increase learning opportunities, we know little about how social information use varies across predator species.
2. Social transmission of avoidance among predators also has potential consequences for defended prey. Conspicuous aposematic prey are assumed to be an easy target for naïve predators, but this cost may be reduced if multiple predators learn by observing single predation events. Heterospecific information use by predators might further benefit aposematic prey, but this remains untested.
3. Here we test conspecific and heterospecific information use across a predator community with wild‐caught blue tits (*Cyanistes caeruleus*) and great tits (*Parus major*). We used video playback to manipulate social information about novel aposematic prey and then compared birds’ foraging choices in ‘a small‐scale novel world’ that contained novel palatable and aposematic prey items.
4. We expected that blue tits would be less likely to use social information compared to great tits. However, we found that both blue tits and great tits consumed fewer aposematic prey after observing a negative foraging experience of a demonstrator. In fact, this effect was stronger in blue tits compared to great tits. Interestingly, blue tits also learned more efficiently from watching conspecifics, whereas great tits learned similarly regardless of the demonstrator species.
5. Together, our results indicate that social transmission about novel aposematic prey occurs in multiple predator species and across species boundaries. This supports the idea that social interactions among predators can reduce attacks on aposematic prey and therefore influence selection for prey defences.

## Graphical abstract

The authors demonstrate that predators use both conspecific and heterospecifics information about prey defences, and this reduces predation on novel aposematic prey. Observing the foraging events of other predator species increases opportunities for social learning, and this can have important effects on prey survival and evolution.

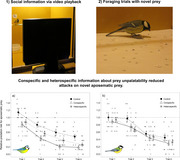

## 1 INTRODUCTION

When encountering potential prey, predators face a decision to either attack or avoid them (Sherratt, 2011). Prey vary considerably in their profitability, such as in their nutrient content and the level of chemical defence (Marples, Speed, & Thomas, 2018; Speed, Ruxton, Mappes, & Sherratt, 2012), and to make adaptive foraging decisions, predators need to gather information about these costs and benefits. In addition to directly interacting with prey, predators can acquire social information about prey profitability by observing the foraging of other individuals (Galef & Laland, 2005). For example, witnessing others consuming palatable food can induce preferences for the same food type (Fryday & Greig‐Smith, 1994; Sherwin, Heyes, & Nicol, 2002). Similarly, predators can acquire social information about prey defences, and this ‘social avoidance learning’ has now been demonstrated in several avian species that avoid unpalatable foods after observing prey rejection (Landová, Svádová, Fuchs, Štys, & Exnerová, 2017) or negative foraging experiences of others (Hämäläinen, Mappes, Rowland, & Thorogood, 2019; Johnston, Burne, & Rose, 1998; Mason, Arzt, & Reidinger, 1984; Mason & Reidinger, 1982; Skelhorn, 2011; Thorogood, Kokko, & Mappes, 2018). Social information about prey defences is assumed to be beneficial for predators, as it can reduce the time and energy invested in prey sampling, as well as the potential costs for consuming toxic prey. However, like many studies of social learning (Danchin, Giraldeau, Valone, & Wagner, 2004; Galef & Laland, 2005), most previous work on social transmission of avoidance has thus far been limited to learning within predator species, and we do not know how social information spreads in a more complex predator community.

Predator communities often consist of several species, therefore providing multiple sources of social information. Predator species may vary in how likely they are to try unpalatable prey and while this is thought to create heterogeneity in selection pressures for prey warning signals (Endler & Mappes, 2004; Nokelainen, Valkonen, Lindstedt, & Mappes, 2014; Valkonen et al., 2012), it may also enhance opportunities for social learning when some predators are more likely to try novel prey than others (Exnerová et al., 2003, 2007). Indeed, the risk of consuming toxins might make social information about prey defences valuable to a broad range of predators, regardless of the identity of the demonstrator. Heterospecific information use has been demonstrated in other contexts (reviewed in Seppänen, Forsman, Mönkkönen, & Thomson, 2007); however, only one previous study has investigated social avoidance learning across predator species (Mason et al., 1984). Predator species might also vary in their response to social information. For example, in contrast to other tested avian species (Hämäläinen et al., 2019; Johnston et al., 1998; Mason et al., 1984; Mason & Reidinger, 1982; Skelhorn, 2011; Thorogood et al., 2018), hens (Sherwin et al., 2002) and blue tits (Hämäläinen, Rowland, Mappes, & Thorogood, 2017) were not found to use social information about food unpalatability in their foraging decisions. However, these previous studies on social avoidance learning all differ in their experimental designs. The strength of the demonstrator's response has been manipulated from the complete avoidance of prey (Landová et al., 2017) to disgust responses ranging from very strong aversive responses, generated by intubating demonstrators with a methiocarb solution (Mason et al., 1984; Mason & Reidinger, 1982), to less severe responses with beak wiping and head shaking after experiencing a bitter taste (Hämäläinen et al., 2019; Johnston et al., 1998; Skelhorn, 2011; Thorogood et al., 2018). Similarly, tests of the effect of social information have varied from choice tests (Hämäläinen et al., 2017; Mason et al., 1984; Mason & Reidinger, 1982) to more complex avoidance learning experiments with novel aposematic prey (Hämäläinen et al., 2019; Landová et al., 2017; Thorogood et al., 2018), making comparison of these different studies challenging.

In addition to potentially benefiting predators, social transmission of information may influence selection for prey defences. Aposematic prey signal their unprofitability to predators with conspicuous warning colouration (Poulton, 1890), but this is only a successful defence once predators have learned to recognize the signal. Until they do, prey face high risks of mortality from uneducated predators (Alatalo & Mappes, 1996; Mappes, Marples, & Endler, 2005). This apparent evolutionary paradox has received wide theoretical and experimental interest, and several different mechanisms might play a role in facilitating the survival of aposematic prey (reviewed in Ruxton, Allen, Sherratt, & Speed, 2018). Predators might, for example, show an increased wariness to attack (Marples, Kelly, & Thomas, 2005; Thomas, Bartlett, Marples, Kelly, & Cuthill, 2004) or handle novel prey (Guilford, 1994), or have an innate aversion to typical warning colours (Lindström, Alatalo, & Mappes, 1999). Aposematic prey are also suggested to benefit from aggregation (Mappes & Alatalo, 1997; Riipi, Alatalo, Lindström, & Mappes, 2001) and kin selection (Mallet & Singer, 1987). Most of this research has, however, focused on how predators learn individually about prey defences, but recent studies suggest that social information use among predators might provide another mechanism favouring the spread of aposematism. Mathematical models (Thorogood et al., 2018) and experiments (Hämäläinen et al., 2019; Thorogood et al., 2018) now show that observational learning by predators can reduce predation risk just enough for novel aposemes to reach fixation in a prey population. Heterospecific social avoidance learning could further increase the strength of this selection, but how common this is across predator communities remains untested.

Here we investigated how wild blue tits and great tits use conspecific and heterospecific information when learning about prey defences. Outside the breeding season, parid tits form mixed species foraging flocks (Ekman, 1989), which provide good opportunities for social learning within and between species. Great tits have been shown to use conspecific information about both palatable (Marchetti & Drent, 2000) and unpalatable food (Hämäläinen et al., 2019; Landová et al., 2017; Thorogood et al., 2018) and novel foraging skills have been found to spread through wild great tit populations (Aplin et al., 2015). In addition, great tits have been demonstrated to use heterospecific information (Brodin & Urhan, 2014), although there is evidence that adult birds are more likely to learn a novel foraging task from a conspecific than from a heterospecific (blue tit) demonstrator (but this difference was not observed in juveniles; Sasvári, 1979, 1985). In blue tits, the evidence for social information use is less clear. Aplin, Sheldon, and Morand‐Ferron (2013) demonstrated that blue tits used social information to learn a novel foraging task, but individuals varied with only approximately 50% of birds learning the task by observing others. In our recent research, we also found no evidence of blue tits using social information in their foraging decisions (Hämäläinen et al., 2017; Hämäläinen, Rowland, Mappes, & Thorogood, 2019). This indicates that the two species might differ in their information use, providing an interesting paradigm to study social learning across a predator community.

Research comparing information use between blue tits and great tits in the same experimental set‐up is, however, limited. To date, the best evidence comes from experiments by Sasvári (1979, 1985) who found that adult great tits were more likely to learn a novel foraging skill socially, compared to adult blue tits (Sasvári, 1979), whereas there was no difference in social learning between juveniles of the two species (Sasvári, 1985). In addition, cross‐fostering experiments in the wild have provided evidence that both species acquire social information about prey types and foraging niches from their parents, but this effect might be stronger in great tits (Slagsvold & Wiebe, 2007, 2011). Experiments with wild parid tit populations have also demonstrated heterospecific information use (Farine, Aplin, Sheldon, & Hoppitt, 2015). Farine et al. (2015) showed that blue tits and great tits acquired information about novel foraging sites from both conspecifics and heterospecifics, but association strengths among heterospecifics were found to be weaker than among conspecifics, suggesting faster information transfer within species. These studies, however, have all investigated how birds learn about positive foraging experiences of others, and we do not know how parid tits differ in their use of social information about unpalatable prey.

We presented blue tits and great tits with social information using video playback of a demonstrator bird (blue tit or great tit) responding to novel aposematic prey. When tasting unpalatable food, birds usually perform vigorous beak wiping and head shaking (Clark, 1970; Hämäläinen et al., 2017; Rowland, Parker, Jiang, Reed, & Beauchamp, 2015) which can provide information for others (Hämäläinen et al., 2019; Johnston et al., 1998; Skelhorn, 2011; Thorogood et al., 2018). Video playback has been used previously with both blue tits (Hämäläinen et al., 2017, 2019) and great tits (Hämäläinen et al., 2019; Smit & van Oers, 2019; Snijders, Naguib, & Oers, 2017; Thorogood et al., 2018), and it provides a good method to control the information that is presented to observers. In both species, we had three treatment groups that received either (a) conspecific or (b) heterospecific information about novel aposematic prey, or (c) saw a control video with prey items only, providing no information of their palatability. We then conducted foraging trials in ‘a small‐scale novel world’ that contained cryptic palatable and conspicuous aposematic prey that were evolutionary novel to the birds (Alatalo & Mappes, 1996; Hämäläinen et al., 2019). We investigated whether receiving social information influenced birds’ foraging choices, and whether information use differed between the species or depended on the demonstrator's identity. As great tits have been found to be more sensitive to social information (Sasvári, 1979; Slagsvold & Wiebe, 2007), we predicted that (a) social information would reduce attacks on the aposematic prey by both species, but (b) great tits would rely on social information more than blue tits, that is, social information would reduce predation risk for aposematic prey more in great tit treatments. Because parid tits have been demonstrated to learn more efficiently from conspecifics (Farine et al., 2015; Sasvári, 1979), we also predicted that (c) individuals would rely more on conspecific information compared to heterospecific information and therefore learn to avoid aposematic prey faster after observing conspecifics.

## 2 MATERIALS AND METHODS

### 2.1 Birds and housing

The experiment was conducted at the Konnevesi Research Station in Central Finland from October to December 2017. We tested 39 great tits (7 female juveniles, 12 male juveniles, 8 female adults and 12 male adults) and 48 blue tits (19 juveniles and 29 adults). Birds were caught from the feeding site in Konnevesi and housed individually in indoor plywood cages (80 × 65 × 50 cm), with a daily light period of 12.5 hr. Fresh water and food (sunflower seeds, tallow and peanuts) were provided ad libitum, except during training and the experiment when food restriction was necessary to motivate birds to forage. After the experiment (approximately 1 week), birds were ringed and released at their capture site. They were weighed (after capture and before the release) and their wing and tarsus lengths were measured. Both species were aged based on their plumage and great tits were sexed (Svensson, 1992). We also classified blue tits to males and females based on their morphological measurements and plumage, but because genetic samples are required to sex the species confidently, we did not include this measure in any of the analyses.

### 2.2 Prey items

We used ‘a small‐scale novel world’ method (Alatalo & Mappes, 1996; Hämäläinen et al., 2019) to investigate the predation risk of novel palatable and aposematic prey. Prey items were small pieces (approximately 0.1 g) of almond flakes that were glued inside a white paper packet (8 × 8 mm) using non‐toxic UHU glue. We used two black symbols (printed on both sides of paper packets) to indicate prey palatability. Palatable prey had a cross symbol that was cryptic to the background, whereas aposematic prey were printed with a conspicuous square symbol. Aposematic prey were made distasteful by soaking almond pieces in bitter‐tasting chloroquine phosphate solution (2 g of chloroquine in 30 ml of water) for 1 hr (e.g. Lindström, Alatalo, Lyytinen, & Mappes, 2001).

Previous studies have shown that great tits do not have a preference for a cross or square symbol (Hämäläinen et al., 2019; Lindström et al., 2001). We followed the same protocol to investigate initial preference in blue tits using 10 individuals that did not participate in the main experiment (see Supporting Information for details of the preference test). We found that when given a choice between a cross and a square symbol (both palatable), blue tits preferred squares. This strong initial preference for squares means that it might be more difficult to detect an effect of social avoidance learning (acquiring avoidance to squares) in blue tits compared to great tits that do not have preferences towards the symbols (Hämäläinen et al., 2019; Lindström et al., 2001). However, it also means that finding an effect of social information use would provide even stronger evidence of social avoidance learning, as it would indicate that birds switched their initial preferences after observing others.

### 2.3 Experimental set‐up

The foraging trials were conducted in a 50 × 66 × 49 cm sized wooden cages that had the front wall made of plexiglass, enabling us to observe birds during the experiment. In each trial, we presented birds novel world backgrounds that contained eight cryptic palatable prey items (cross symbol) and eight conspicuous aposematic prey items (square symbol). Backgrounds were made of A1‐sized white paper sheets that had 140 crosses printed in random positions to make palatable prey cryptic. To increase the difficulty to find cryptic prey, we also made the background three‐dimensional by adding in each sheet 20 fake cryptic prey items (a piece of double‐sided mounting tape with a cross symbol), following previously established methods (e.g. Hämäläinen et al., 2019; Lindström et al., 2001). Backgrounds were covered with adhesive plastic, and prey items (eight of each type) were randomly distributed and glued to them.

Previous studies have tested symbol visibility with great tits, showing that squares are approximately four times more visible against the background in a large aviary (Lindström, Alatalo, Mappes, Riipi, & Vertainen, 1999) and in our ‘small‐scale novel world’ set up (Hämäläinen et al., 2019). Before the main experiment, we conducted the visibility test with blue tits, using the same 10 individuals that were tested for symbol preference (see Supporting Information for details). Birds were required to consume 20 prey, and similar to great tits (Hämäläinen et al., 2019; Lindström et al., 1999), blue tits were found to consume more squares than crosses (on average 15 squares and 5 crosses), which suggests that squares are more visible against the background. However, because blue tits also preferred squares before the visibility test, it is difficult to disentangle this preference from the visibility of the symbols.

### 2.4 Video playback

Birds were provided social information using video playback of a foraging demonstrator (conspecific/heterospecific). We filmed four adult great tits and four adult blue tits as demonstrators for the videos. To reduce variation among demonstrations, all demonstrators were males (although the sex of blue tits could not be determined with 100% confidence without genetic sampling). Some of the demonstrators (all blue tits and one great tit) participated in the experiment also as observers, and they were filmed as demonstrators for others after they had finished the avoidance learning trials. Demonstrators’ responses to aposematic prey were filmed through the plexiglass wall of the cage with an HD camcorder (Canon Legria HF R66), following previously established methods (Hämäläinen et al., 2019; Thorogood et al., 2018). An aposematic prey item was similar to the prey used in the main experiment (a square symbol) but bigger in size (20 × 20 mm) to ensure that it was visible to observers.

We filmed a demonstrator taking the aposematic prey item from the cage floor, opening it on the perch and tasting it. Following this, birds showed a clear disgust response by wiping their beak on the perch and shaking their head. The length of these responses varied among demonstrators and we aimed to standardize their strength by editing the videos (using Windows Movie Maker) so that they all consisted of 80 s of a demonstrator's response to aposematic prey (see Supporting Information for details about variation among videos). Videos also included 80 s of an alternative prey with a cross symbol in an empty cage (40 s before and 40 s after a demonstrator) to make sure that birds had seen both prey types before the foraging trials and the familiarity of symbols would not influence their preferences. We filmed and edited eight different videos (one of each demonstrator) and each video was used in six demonstrations (for three blue tit and three great tit observers). In addition, we recorded a control video that showed only prey items in an empty cage (80 s each). This was presented to the control groups that did not receive information about prey palatability. A demonstrator bird was not included in control videos, as this could have provided observers unintended social information of the demonstrator rejecting the prey via avoidance.

### 2.5 Foraging trials

Before the experiment, birds were trained to consume artificial prey items, following previously established methods (e.g. Hämäläinen et al., 2019; Lindström et al., 2001). The first training phase was done in home cages, where birds were trained to open brown paper packets and to detach them off the training background that was printed with >? symbols. During training, birds did not have access to other food (for detailed methods, see Hämäläinen et al., 2019). The last training phase was conducted in the test cage on the same day when the experiment started. We presented birds a training background that resembled the backgrounds that they later encountered in the foraging trials (i.e. with cross symbols). This background contained three brown and three cryptic (cross) prey items and we waited for birds to find and eat all of them before starting the experiment. The same protocol has been used in previous experiments (Hämäläinen et al., 2019; Thorogood et al., 2018) and it ensures that birds learn to forage in the novel world environment before the learning trials. Other food was restricted for 2 hr before the experiment to ensure birds’ motivation to forage.

After birds had completed the last training phase (i.e. consumed three brown and three cryptic prey), we provided them information about aposematic prey via video playback. In both species, individuals were randomly allocated to three treatments that (a) received social information from a conspecific (*N* = 12 in both species), (b) received social information from a heterospecific (*N* = 12 in both species) or (c) did not receive any social information (control group) before four foraging trials (great tits: *N* = 12; blue tits: *N* = 14). Two blue tits in the control group completed only the first trial, with one of them refusing to consume any prey in the second trial, and another one getting injured (this was not related to the experiment, and the bird recovered and was released afterwards). Therefore, the blue tit control group includes 14 individuals that completed the first trial and 12 individuals that completed all four trials.

**Figure 1:**
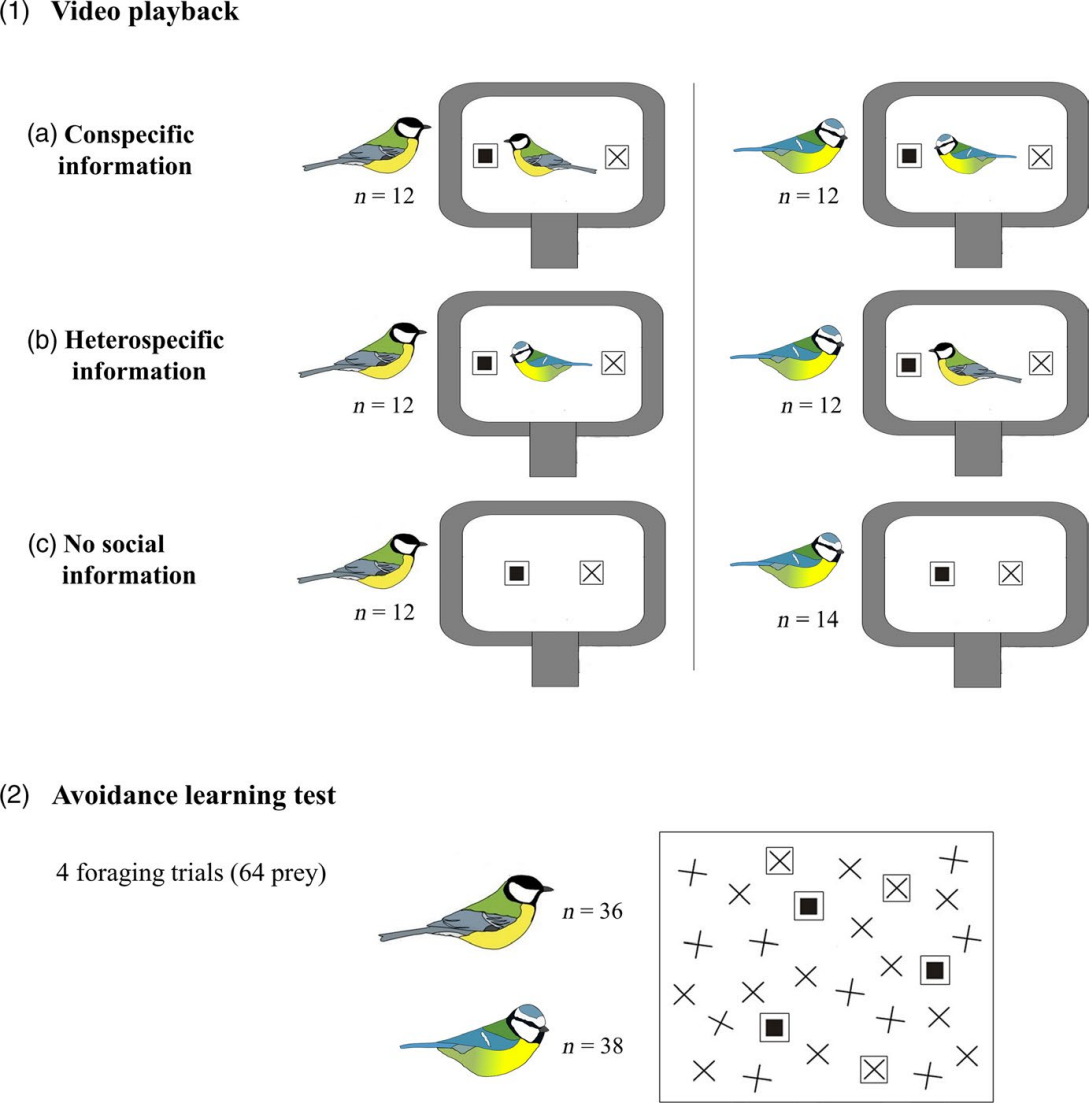
Experimental set‐up. Great tits and blue tits were first presented with video playback of (a) a conspecific or (b) a heterospecific attacking a novel aposematic prey (prey with a square symbol) and an alternative prey (a cross symbol) in an empty cage, or (c) prey items only (control group with no information about prey palatability). We then conducted an avoidance learning test in ‘a small‐scale novel world’, where birds encountered cryptic palatable prey (crosses) and conspicuous aposematic prey (squares). We investigated avoidance learning across four foraging trials (conducted over two consecutive days), in each of which birds were allowed to consume 16 prey items

Video playback was shown from a computer monitor (Dell E198FPF) that was positioned against the plexiglass wall of the cage. Birds were first allowed to habituate to the monitor for 15 min, and then presented a video of a conspecific or a heterospecific demonstrator, or only the prey (Figure 1). Even though these videos were not capturing the UV cues in birds’ plumage, observers were likely to recognize conspecifics and heterospecifics easily based on other species‐specific visual characteristics (such as plumage patterns). Immediately after the video, the monitor was removed and birds were presented a first novel world background that contained eight palatable crosses and eight aposematic squares. Birds were allowed to consume four prey items before the background was removed and replaced with a new one. In each trial, birds were sequentially presented four different backgrounds, allowing them to consume 16 prey items in total (four from each background). If birds failed to consume all four prey items during the first 20 min, we removed the background and waited for birds to be more motivated to forage before continuing the trial with the same background.

We conducted two foraging trials on the first day of the experiment (with at least 30 min break between the trials) and two trials on a following day. Birds were not provided with further social information on the second day to investigate whether the effect of social information persisted across days. During the experiment, birds consumed in total 64 prey items (16 in each of the four trials) and we recorded their foraging choices. In addition, we recorded how fast birds started the first foraging trial to see whether this was influenced by received social information. Sometimes, birds took the prey from the background but did not open them. We did not count this as consumption of the prey because birds did not taste the prey items and therefore did not receive any information about their palatability. Previous studies have also demonstrated that aposematic insects often survive an encounter with avian predators (Exnerová et al., 2003; Umbers et al., 2019; Wiklund & Järvi, 1982), and we assumed that picking up the prey without further handling would not ‘kill’ it. Nevertheless, handling by predators might have some fitness costs for prey. To investigate how social information influenced these costs, we additionally recorded the first 16 prey items that birds handled during the first trial, regardless of whether they opened them or not.

### 2.6 Statistical analyses

We first tested whether birds’ first foraging choice depended on received social information using a chi‐square test. Differences in the latency to start foraging (i.e. to attack the first prey item) were then analysed using a Cox regression model, as the response variable (time before attacking the first prey item) was time before an event type. This was explained by the interaction between information treatment (conspecific/heterospecific/control) and species (blue tit/great tit), and individuals' age and body condition index as covariates. Body condition index was assumed to indicate individuals' energetic reserves and it was calculated for each individual based on the relationship of their weight and tarsus measures (Peig & Green, 2009). Because of the different size of blue tits and great tits, we calculated body condition index separately for each species and then scaled these values with the mean and standard deviation to get a body condition measure that was comparable across the species. We did not have a tarsus measurement from one great tit (in heterospecific treatment) and this individual was therefore excluded from the models that included body condition index.

Differences in the number of aposematic prey consumed or handled during the foraging trials were analysed using generalized linear models with a binomial error distribution (logit link function), with the number of aposematic and palatable prey consumed or handled as a bound response variable. We first analysed birds’ foraging choices in the first foraging trial after video playback. Explanatory variables in the model included an interaction between information treatment and species, and individuals' age and body condition index as covariates. We then analysed how birds improved across four foraging trials. The number of aposematic and palatable prey consumed in each trial was used as a bound response variable and this was explained by information treatment, species and trial number that was included as a continuous variable (trials 1–4). We started the model selection with the model that included a three‐way interaction between the explanatory variables and assessed the significance of the terms in the model using likelihood ratio tests (Table 1). All models included age and body condition index as covariates and bird identity as a random effect. The analyses were conducted in r version 3.6.1 (R Core Team, 2019), using lme4 (Bates, Mächler, Bolker, & Walker, 2015) and survival packages (Therneau, 2015).

**Table 1:** Generalized linear model explaining the number of aposematic prey that birds (*n* = 74) (a) handled and (b) consumed in the first trial (first 16 prey items). Intercept gives the estimate (logit) for the aposematic prey that adult blue tits handled or consumed when they did not receive social information (control group)

Terms in the model	Effect	*SE*	*Z*	*p*
(a) Prey handled				
Intercept	0.268	0.118	2.277	.02
Conspecific information	−0.436	0.145	−2.998	.003
Heterospecific information	−0.315	0.144	−2.188	.03
Species (great tit)	−0.006	0.120	−0.047	.96
Age (juvenile)	0.072	0.123	0.587	.56
Body condition	−0.005	0.060	−0.075	.94
(b) Prey consumed				
Intercept	0.149	0.117	1.275	.20
Conspecific information	−0.460	0.145	−3.165	.002
Heterospecific information	−0.318	0.144	−2.213	.03
Species (great tit)	0.002	0.120	0.014	.99
Age (juvenile)	0.012	0.123	0.095	.92
Body condition	−0.001	0.060	−0.017	.99

## 3 RESULTS

### 3.1 First foraging trial

**Figure 2:**
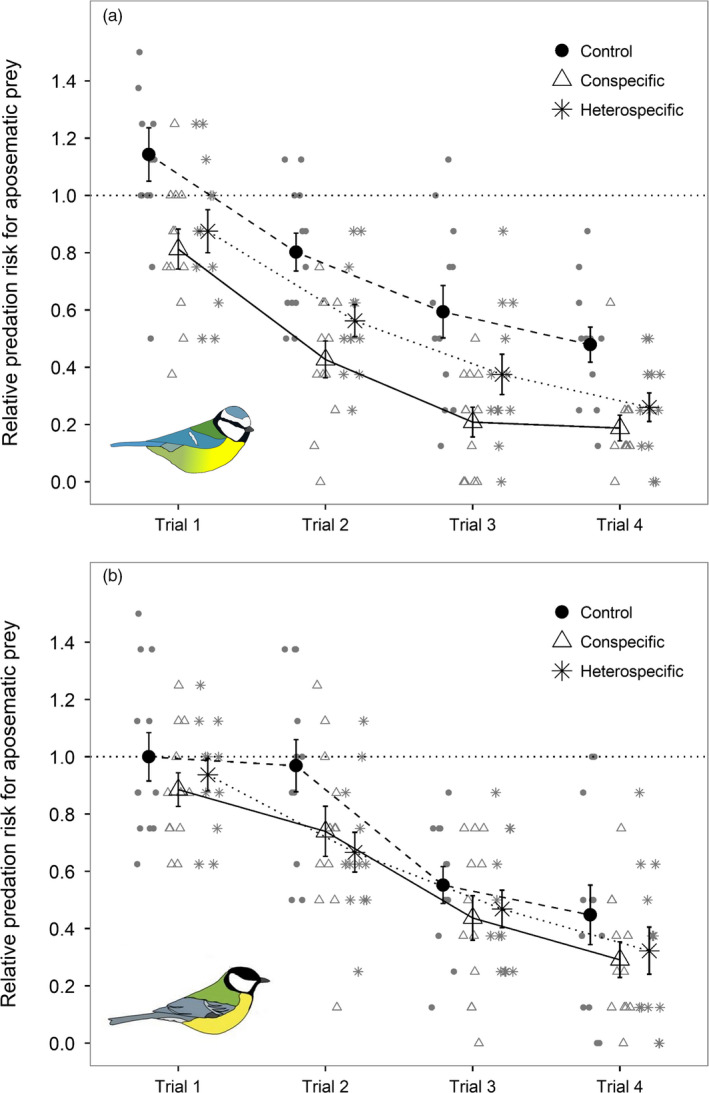
Relative predation risk (mean ± *SE*) for aposematic prey (number of aposematic prey consumed/ number expected by random chance) with (a) blue tit and (b) great tit predators. The graph shows the decrease in predation risk over four trials that were conducted over two consecutive days (two trials/day). Each species had three treatment groups that (i) did not receive any social information (circles + dashed lines), (ii) received social information about aposematic prey from a conspecific (triangles + solid line) or (iii) received social information about aposematic prey from a heterospecific (stars + dotted line). Smaller symbols indicate individual variation within the treatment

We found that social information affected how both blue tits and great tits responded to the prey during their initial encounter. While social information treatment did not influence which prey item great tits (chi‐square test: *χ* ^2^ = 0.892, *df* = 2, *p* = .64) or blue tits (chi‐square test: *χ* ^2^ = 0.829, *df* = 2, *p* = .66) attacked first, it reduced the overall predation risk for aposematic prey during the first trial (Figure 2). We found that birds handled and consumed fewer aposematic prey after receiving either conspecific or heterospecific information (Table 1). This decrease was biologically important, as it reduced the predation risk for aposematic prey below 1.0 (Figure 2), therefore altering the relative fitness of aposematic and cryptic prey phenotypes. There were no differences between the two social information treatments in the number of aposematic prey handled (compared to conspecific information, the effect of heterospecific information = 0.120 ± 0.148, *Z* = 0.815, *p* = .42) or consumed (compared to conspecific information, the effect of heterospecific information = 0.142 ± 0.149, *Z* = 0.956, *p* = .34). The effect of social information was similar in both species (information treatment × species [a] handling: *χ* ^2^ = 1.675, *df* = 2, *p* = .43 and [b] consumption: *χ* ^2^ = 3.446, *df* = 2, *p* = .18). Furthermore, the number of aposematic prey handled or consumed in the first trial did not differ between the species, and nor was it affected by either individuals’ age or body condition (Table 1). Most birds attacked the first prey item in the experiment quickly (median = 44 s, range = 4–3286 s); however, seven birds were considerably slower than others and took longer than 5 min to begin the experiment. This latency was not explained by conspecific (compared to control group, the effect of conspecific information = 0.158 ± 0.311, *Z* = 0.506, *p* = .61) or heterospecific information (compared to control group, the effect of heterospecific information = 0.376 ± 0.306, *Z* = 1.228, *p* = .22), but we found that blue tits tended to attack the first prey faster than great tits (the effect of species [great tit] = −0.411 ± 0.244, *Z* = −1.687, *p* = .09; see Supporting Information for the full model).

### 3.2 Learning across trials

Both blue tits and great tits showed increased avoidance of the aposematic prey over the course of the experiment (effect of trial number = −0.542 ± 0.032, *Z* = −17.084, *p* < .001; Figure 2). However, while there were no differences in learning rate between the species according to information treatment (Table 2), blue tits and great tits responded to the source of social information differently overall (compared to blue tit control group, the effect of conspecific information × species = 0.509 ± 0.234, *Z* = 2.177, *p* = .03). To investigate these differences further, we next ran separate models for each species.

**Table 2:** Comparison of GLMMs explaining the number of aposematic prey consumed during the four foraging trials. Abbreviations of the explanatory variables are as follows: S = species (blue tit/great tit), I = information treatment (conspecific/heterospecific/control), T = trial number (1–4), C = body condition, A = age, ID = bird identity. We started model selection with a model that included a three‐way interaction between species, information treatment and trial number, and removed the interaction terms based on their significance

Model	Model *df*	AIC	*χ^2^*	*df*	*p*
~ S*I*T + C + A + 1|ID	15	1,188.4			
~ S*I + I*T + S*T + C + A + 1|ID	13	1,185.5	1.040	2	.59
~ S*I + I*T + C + A + 1|ID	12	1,184.8	1.302	1	.25
~ S*I + C + A + 1|ID	10	1,183.6	2.832	2	.24

In blue tits, both conspecific and heterospecific information about prey unpalatability reduced predation risk for aposematic prey (Table 3a; Figure 2a). This pattern was similar in great tits, although the effect of social information was not significant at alpha level 0.05 (Table 3b; Figure 2b). However, seven individuals had a very high initial wariness to consume novel prey (latency to start the experiment >5 min). Five of these birds were great tits (two in conspecific information and three in control treatment), and the effect of social information was stronger when these outliers were excluded from the analysis (effect of conspecific information = −0.358 ± 0.186, *Z* = −1.923, *p* = .05; effect of heterospecific information = −0.355 ± 0.181, *Z* = −1.960, *p* = .05). In blue tits, social information from conspecifics reduced predation on aposematic prey even more than social information from heterospecifics (compared to conspecific information, the effect of heterospecific information = 0.322 ± 0.159, *Z* = 2.023, *p* = .04; Figure 2a). We did not, however, detect this difference in source of social information in great tits (effect = 0.035 ± 0.174, *Z* = 0.202, *p* = .84; Figure 2b). Finally, we found that age and body condition influenced great tits' foraging choices across the experimental trials, with adults and birds in a poor body condition consuming more aposematic prey (Table 3b), whereas we found no evidence that age or body condition influenced the blue tits’ tendency to consume aposematic prey (Table 3a).

**Table 3:** Generalized linear mixed effects models explaining the number of aposematic prey that (a) blue tits (*n* = 36) and (b) great tits (*n* = 36) consumed during the experiment (across four foraging trials). Intercept gives the estimate (logit) for the aposematic prey that adult birds consumed in the first trial when they did not receive social information (control group)

Terms in the model	Effect	*SE*	*Z*	*p*
(a) Blue tits				
Intercept	0.305	0.117	2.600	.009
Conspecific information	−0.926	0.155	−5.982	<.001
Heterospecific information	−0.603	0.145	−4.169	<.001
Trial number	−0.573	0.046	−12.542	<.001
Age (juvenile)	−0.005	0.135	−0.038	.97
Body condition	−0.035	0.061	−0.574	.57
(b) Great tits				
Intercept	0.372	0.166	2.240	.03
Conspecific information	−0.311	0.169	−1.836	.07
Heterospecific information	−0.276	0.170	−1.621	.11
Trial number	−0.513	0.044	−11.653	<.001
Age (juvenile)	0.388	0.139	−2.790	.005
Sex (male)	−0.042	0.140	−0.302	.76
Body condition	−0.170	0.072	−2.374	.02

## 4 DISCUSSION

Social information about prey defences can influence predators' foraging decisions and reduce predation on novel aposematic prey (Hämäläinen et al., 2019; Johnston et al., 1998; Landová et al., 2017; Mason & Reidinger, 1982; Thorogood et al., 2018). However, experiments comparing ecologically similar predator species that have potential to learn from one another's foraging behaviour are scarce (e.g. Lefebvre, Templeton, Brown, & Koelle, 1997; Mason et al., 1984; May & Reboreda, 2005; Sasvári, 1979). Here we combine these in one experiment to test the effects of conspecific and heterospecific information on avoidance learning of two predator species. We found that both blue tits and great tits used social information about prey unpalatability and that this reduced predation pressure on novel aposematic prey. Importantly, we also showed that both species could learn by observing each other. Although we expected that blue tits may not use social information as much as great tits (Aplin et al., 2013; Hämäläinen et al., 2017, 2019; Sasvári, 1979), surprisingly we found the opposite. Blue tits consumed fewer aposematic prey after observing a conspecific or a heterospecific demonstrator attacking the same prey signal. The trend was similar in great tits, although the effect was less clear than in our previous studies with a similar set‐up (Hämäläinen et al., 2019), or conducted at a larger scale where foraging costs may have differed (Thorogood et al., 2018). Our study suggests that social transmission about novel prey signals can occur across and among predator species and it could therefore have potent effects on prey evolution.

Social learning theories predict that individuals should value social information more when the cost to obtain personal information is high (Kendal, Coolen, Bergen, & Laland, 2005; Laland, 2004). Therefore, social information about unpalatable food is likely to be important to predators if it prevents them ingesting potentially toxic food, and it might even be more valuable than information gathered from observing palatable foraging experiences. This could explain why we found strong evidence of blue tits learning by observing others in this experiment, in contrast to previous studies that focused on solving a foraging task (Aplin et al., 2013; Sasvári, 1979), or using social information in a simple multi‐choice foraging test (Hämäläinen et al., 2017, 2019). In our current experiment, birds encountered a more complex foraging environment where they were required to attack many novel prey and the higher energy and time investment, together with the risk of consuming prey with unknown toxin quantity might have increased the relative costs of gathering personal information (Skelhorn, Halpin, & Rowe, 2016). Furthermore, our experiment also demonstrates that blue tits can learn by watching video playback of a demonstrator. This is in contrast to our earlier work that suggested blue tits do not necessarily use the information provided, even though they paid attention to video playback of a foraging conspecific (Hämäläinen et al., 2017). This indicates that social information use is context‐dependent, and the failure to find clear evidence of the efficacy of videos in our previous studies resulted from different methods such as simpler foraging tasks (Hämäläinen et al., 2017, 2019), highlighting the importance of standardized experiments to compare information use across species (Shaw & Schmelz, 2017).

Our results suggest that social transmission is an important part of predator learning. However, predators may observe many different behaviours of other individuals, and we do not know which of these are the most salient signals of prey unprofitability. For example, visible disgust responses after experiencing unpalatable food, like those presented in our videos, are common across species (Brooke, 2019) and potentially provide an important cue about prey defences (Skelhorn, 2011). However, it has also been demonstrated that great tits can learn about aposematic prey by observing an educated conspecific ignoring it (Landová et al., 2017). While our control video (prey items only) ensured that predators had no social information available, it unfortunately does not allow us to distentangle which aspect of a demonstrator's behaviour shifted observers’ foraging decisions away from aposematic prey. The simple presence of another individual may influence food choices through competition (McMahon, Conboy, O'Byrne‐White, Thomas, & Marples, 2014), and it is possible that birds in our experiment preferred a different prey symbol than a demonstrator to avoid potential competitors. Nevertheless, previous work with blue tits suggests that observers pay more attention to negative (i.e. visible cues of head shaking and beak wiping) than positive (i.e. consumption) foraging events (Hämäläinen et al., 2017), and domestic chicks show stronger biases in their foraging choices when demonstrators perform more beak wipes and head shakes (Skelhorn, 2011). In our experiment, all demonstrators performed very strong responses, as we aimed to standardize the presented information. To better understand how a demonstrator's behaviour influences avoidance learning, future work should therefore manipulate the strength of the disgust response to investigate how this affects the foraging choices of predators observing the event.

Previous studies have suggested that social avoidance learning can help facilitate the initial evolution of aposematic prey (Hämäläinen et al., 2019; Thorogood et al., 2018). Our finding that species can also learn from observing each other further supports this hypothesis as it increases both the potential audience and the availability of demonstrators. However, as in previous studies (Hämäläinen et al., 2019; Thorogood et al., 2018), we found that both blue and great tits varied in their strength of response to social information, with some individuals sampling more aposematic prey than others. Furthermore, we show that blue tits learned more from observing conspecifics than heterospecifics, whereas great tits did not show a bias according to demonstrator species, despite previous experiments suggesting that great tits learn novel foraging skills better from observing conspecifics (Sasvári, 1979). This variation among and within predator species in how they rely on different social information sources to access or attack prey could have important consequences. For example, when some individuals continue to try new prey for longer, this provides additional opportunities for others to learn, including naïve immigrants and juveniles (Mappes, Kokko, Ojala, & Lindström, 2014; Thorogood et al., 2018). Or, if predator species vary in their neophobia (e.g. blue tits are more hesitant to attack novel prey than great tits; Adamová‐Ježová, Hospodková, Fuchsová, Štys, & Exnerová, 2016; Exnerová et al., 2007), then social information from heterospecifics might be an important source of information for the more neophobic species. This variation among predators would create varying selection pressures for warning signals in space and time (Endler & Mappes, 2004; Thorogood et al., 2018), perhaps favouring more conspicuous warning signals when predator communities are more likely to learn about aposematic prey socially. Variation in social information use among predators could therefore help to maintain signal polymorphisms in the face of frequency‐dependent selection (Nokelainen et al., 2014), as well as influence the cost of signal conspicuousness (Valkonen et al., 2012).

Heterospecific information use has been now demonstrated in many different contexts (Seppänen et al., 2007). For example, Carib grackles copy the foraging techniques from both conspecific and heterospecific (zenaida dove) demonstrators (Lefebvre et al., 1997), and shiny cowbirds learn a novel foraging task after observing either a conspecific or a heterospecific (a screaming cowbird; May & Reboreda, 2005). Our study extends this growing body of evidence and shows that social avoidance learning occurs in multiple predator species and across species boundaries. Although there is some evidence that primates can also learn about unpalatable food socially (Snowdon & Boe, 2003; Van de Waal, Borgeaud, & Whiten, 2013), ‘social avoidance learning’ is yet to be tested in more than a handful of (avian) predator species (Johnston et al., 1998; Landová et al., 2017; Mason & Reidinger, 1982; Thorogood et al., 2018), or outside of highly controlled conditions in captivity. The situation is likely to be more complicated in the wild where predators can encounter many different prey types and have opportunities to gather social information from both conspecifics and heterospecifics. Field studies across different predator communities are therefore required to increase our understanding of social transmission in predator populations and its effects on predator–prey coevolution.

## AUTHORS’ CONTRIBUTIONS

L.H., H.M.R., J.M. and R.T. conceived and designed the experiment. L.H. and M.T. collected the data. L.H. and R.T. analysed the data. L.H. led the writing of the manuscript. All authors contributed critically to the drafts and gave final approval for publication.

## Data Availability

All data are available from the University of Cambridge Data Repository: https://doi.org/10.17863/CAM.47021 (Hämäläinen, Mappes, Rowland, Teichmann, & Thorogood, 2019).
