# Supplementary material for: Social learning within and across predator species reduces attacks on novel aposematic prey
Source: J Anim Ecol. 2020 Feb 19;89(5):1153–64. doi: 10.1111/1365-2656.13180 (PMC7317195; doi:10.1111/1365-2656.13180)
Supplement: Supplementary file 1 [file JANE-89-1153-s001.docx]

**Supplementary material for**

**Social learning within and across predator species reduces attacks on novel aposematic prey**

**Liisa Hämäläinen^1^, Johanna Mappes^2^, Hannah M. Rowland^1,3,4^, Marianne Teichmann^5,6,7^ & Rose Thorogood^1,5,6^**

^1^Department of Zoology, University of Cambridge, UK

^2^Department of Biological and Environmental Sciences, University of Jyväskylä, Finland

^3^Max Planck Institute for Chemical Ecology, Jena, Germany

^4^Institute of Zoology, Zoological Society of London, UK

^5^HiLIFE Helsinki Institute of Life Science, University of Helsinki, Finland

^6^Research Programme in Organismal & Evolutionary Biology, Faculty of Biological and Environmental Sciences, University of Helsinki, Finland

^7^Chair of Nature Conservation & Landscape Ecology, University of Freiburg, Germany

**Correspondence:**

Liisa Hämäläinen

llh35@cam.ac.uk

**1. SUPPLEMENTARY METHODS**

**1.1 Prey symbol preference and visibility tests**

Before conducting the learning trials, we tested whether blue tits had initial preferences towards the prey symbols, following similar methods as previous preference tests in great tits (Lindström et al. 2001, Hämäläinen et al. 2019). In the preference test blue tits were offered a choice between prey with a cross or a square symbol (both palatable) that were presented simultaneously on a white feeding tray, ensuring that both symbols were equally visible. This was repeated six times and we alternated which symbol was on the left and which on the right side of the tray. Individuals were always allowed to eat both prey items and we recorded their first choice. We then used a generalized linear mixed model with a binomial error distribution to investigate if blue tits preferred either of the symbols. The model included the order in which birds consumed prey items as a response variable and this was explained by prey symbol (cross/square) and bird identity as a random effect. We found that blue tits preferred the prey with a square symbol during the six symbol pair presentations (estimate = -2.023 ± 0.413, Z = -4.900, *P* < 0.001). When the prey items were presented for the first time (i.e. first symbol pair), 8 individuals chose the prey with a square symbol and only 2 individuals the prey with a cross symbol. However, this preference was less clear during the last two symbol pair presentations (6 individuals chose a square and 4 individuals a cross), which suggests that positive experience of both symbols might have reduced birds’ initial preference for squares.

We next tested the visibility of symbols using the same 10 individuals that participated in the preference test. Because each individual had consumed 6 crosses and 6 squares in the preference test, they had equal experience with both symbols and were therefore predicted to attack prey according to the visibility. Birds were required to find and eat 20 prey items from ‘novel world’ backgrounds that contained only palatable prey. Each background contained 8 prey of each symbol type, and birds were presented five backgrounds that were replaced once the bird had taken 4 prey items. We then used a paired sample t-test to compare the number of each prey type consumed. We found that blue tits attacked more squares than crosses (paired samples t-test: t = 7.632, df = 9, *P* < 0.001), consuming on average 15 squares and 5 crosses. This is similar to previous visibility tests in great tits (Lindström et al. 1999; Hämäläinen et al. 2019) and suggests that squares are more visible against the background, although the observed difference might be also explained by blue tits’ initial preference for squares, and disentangling these two effects is difficult.

***References***

Hämäläinen, L., Mappes, J., Rowland, H. M., & Thorogood, R. (2019). Social information use about novel aposematic prey is not influenced by a predator's previous experience with toxins. *Functional Ecology.*

Lindström, L., Alatalo, R. V., Mappes, J., Riipi, M., & Vertainen, L. (1999). Can aposematic signals evolve by gradual change?. *Nature*, 397, 249–251.

Lindström, L., Alatalo, R. V., Lyytinen, A., & Mappes, J. (2001). Predator experience on cryptic prey affects the survival of conspicuous aposematic prey. *Proceedings of the Royal Society of London. Series B: Biological Sciences*, 268, 357–361.

**1.2 Demonstrator videos**

We filmed eight demonstrators (four blue tits and four great tits) to provide observers social information about prey unpalatability. All demonstrators responded to aposematic prey by performing beak wiping and head shaking, but the length of these responses varied across demonstrators. To reduce this variation, the videos were edited to include 80 s of a demonstrator attacking (picking up and opening) an aposematic prey and showing a disgust response. However, the total number of beak wipes on the final video still varied across demonstrators, ranging from 50–120 in blue tits (mean = 73) and from 67–126 in great tits (mean = 86). Similarly, the number of head shakes on the videos varied from 4–22 in blue tits (mean = 9) and from 11–26 in great tits (mean = 19). We tested whether a demonstrator´s behaviour on the video influenced observers’ (n = 48) foraging choices (i) in the first foraging trial (first 16 prey) and (ii) in all four trials (i.e. 64 prey) using a generalized linear model with a binomial error distribution. The number of aposematic and palatable prey attacked was used as a bound response variable and this was explained by the presented number of beak wipes and head shakes. We did not find evidence that the number of beak wipes on the video influenced how many aposematic prey observers attacked during the first trial (estimate = 0.002 ± 0.003, Z = 0.529, *P* = 0.60) or in total during the experiment (estimate = -0.002 ± 0.002, Z = -1.092, *P* = 0.27; Figure 1a). Similarly, the number of head shakes did not influence the tendency to attack aposematic prey in the first trial (estimate = -0.002 ± 0.011, Z = -0.154, *P* = 0.88) or in total (estimate = 0.011 ± 0.006, Z = 1.715, *P* = 0.09; Figure 1b). This suggests that even the videos with the lowest number of head shakes and beak wipes provided observers clear social information about prey unpalatability.


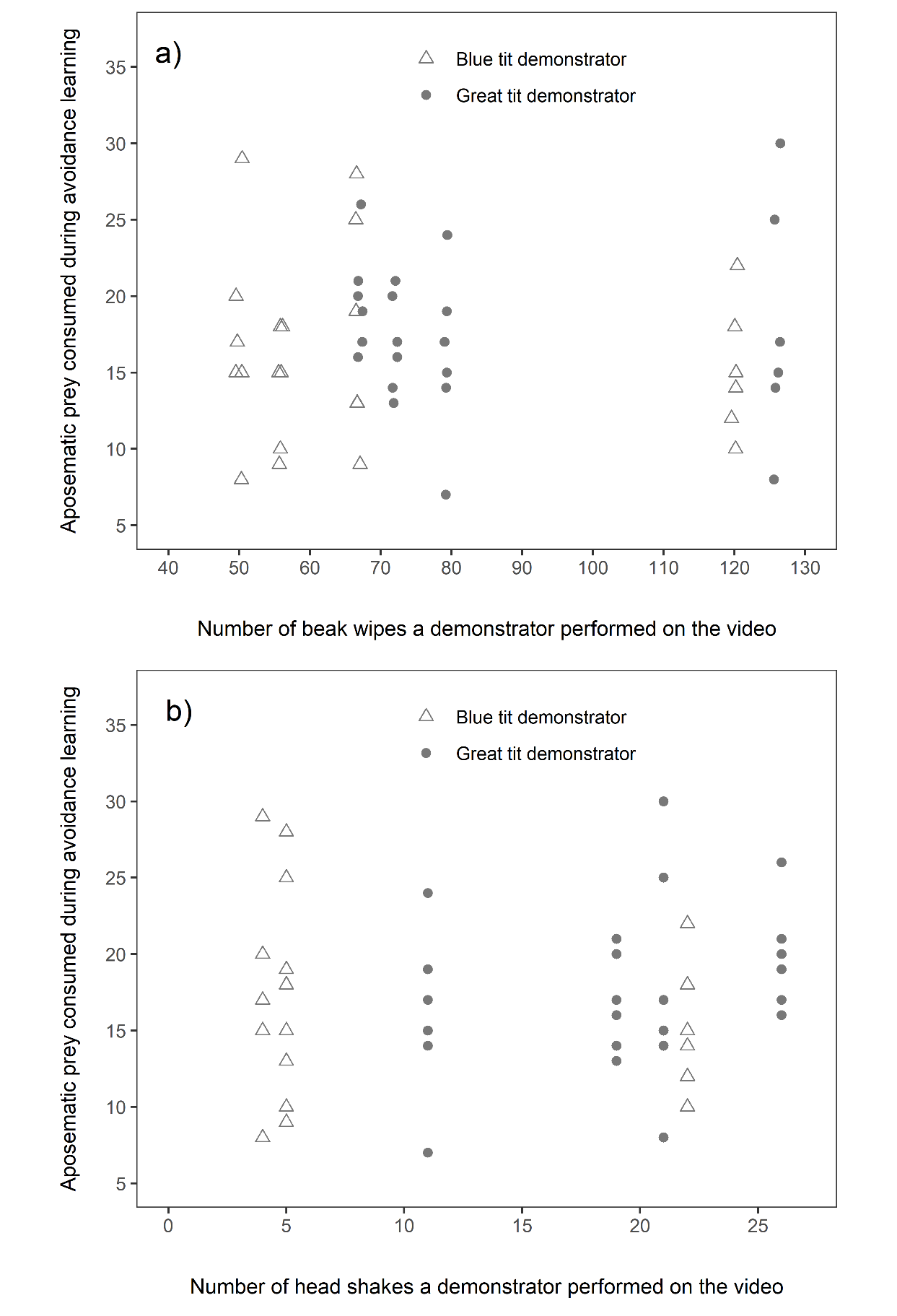


Figure 1. The number of beak wipes (a) or head shakes (b) that a demonstrator performed on the video (80 s) did not influence the total number of aposematic prey that observers (n = 48) consumed during learning trials. Open triangles indicate that the demonstrator was a blue tit and filled circles represent great tit demonstrators.

**2. SUPPLEMENTARY ANALYSIS**

**2.1 Time to attack the first prey item**

Table 1. Cox regression model explaining the latency (s) to attack the first prey item in the experiment (n = 74). The effect of conspecific (compared to blue tit control group, the effect of conspecific information * species = 0.367 ± 0.602, Z = 0.609, *P* = 0.54) or heterospecific information (compared to blue tit control group, the effect of heterospecific information * species = 0.420 ± 0.592, Z = 0.710, *P* = 0.48) did not differ between the species, and this interaction was excluded from the final model.

| Terms in the model | Effect | SE | Z | *P* |
| --- | --- | --- | --- | --- |
| Conspecific information | 0.158 | 0.311 | 0.506 | 0.61 |
| Heterospecific information | 0.376 | 0.306 | 1.228 | 0.22 |
| Species (great tit) | -0.411 | 0.244 | -1.687 | 0.09 |
| Age (juvenile) | 0.162 | 0.260 | 0.625 | 0.53 |
| Body condition | 0.061 | 0.130 | 0.471 | 0.64 |
